# Leaders’ perspectives on learning health systems: a qualitative study

**DOI:** 10.1186/s12913-020-05924-w

**Published:** 2020-11-26

**Authors:** Joanne Enticott, Sandra Braaf, Alison Johnson, Angela Jones, Helena J. Teede

**Affiliations:** 1 Monash Centre for Health Research and Implementation, Monash University, 43-51 Kanooka Grove, Clayton, VIC 3168 Australia; 2 Monash Partners Academic Health Science Centre, 43-51 Kanooka Grove, Clayton, VIC 3168 Australia; 3 School of Public Health and Preventive Medicine, Monash University, 553 St Kilda Road, Melbourne, VIC 3004 Australia

**Keywords:** Learning health system, Informatics, Data-driven healthcare, evidence, healthcare improvement

## Abstract

**Background:**

Integrated utilisation of digital health data has the power to transform healthcare to deliver more efficient and effective services, and the learning health system (LHS) is emerging as a model to achieve this. The LHS uses routine data from service delivery and patient care to generate knowledge to continuously improve healthcare. The aim of this project was to explore key features of a successful and sustainable LHS to inform implementation in an Academic Health Science Centre context.

**Methods:**

We purposively identified and conducted semi-structured qualitative interviews with leaders, experienced in supporting or developing data driven innovations in healthcare. A thematic analysis using NVivo was undertaken.

**Results:**

Analysis of 26 interviews revealed five themes thought to be integral in an effective, sustainable LHS: (1) Systematic approaches and iterative, continuous learning with implementation into healthcare contributing to new best-practice care; (2) Broad stakeholder, clinician and academic engagement, with collective vision, leadership, governance and a culture of trust, transparency and co-design; (3) Skilled workforce, capability and capacity building; (4) Resources with sustained investment over time and; (5) Data access, systems and processes being integral to a sustainable LHS.

**Conclusions:**

This qualitative study provides insights into the elements of a sustainable LHS across a range of leaders in data-driven healthcare improvement. Fundamentally, an LHS requires continuous learning with implementation of new evidence back into frontline care to improve outcomes. Structure, governance, trust, culture, vision and leadership were all seen as important along with a skilled workforce and sustained investment. Processes and systems to optimise access to quality data were also seen as vital in an effective, sustainable LHS. These findings will inform a co-designed framework for implementing a sustainable LHS within the Australian healthcare and Academic Health Science Centre context. It is anticipated that application of these findings will assist to embed and accelerate the use of routine health data to continuously generate new knowledge and ongoing improvement in healthcare delivery and health outcomes.

## Background

There is a growing interest in how best to use evidence and health data to inform decision making in healthcare delivery [1]. Systems are required to ensure that the most relevant and current information is used to guide decisions related to healthcare delivery [2, 3]. Improved health outcomes will require informed decision making at all levels of healthcare, including decisions made by policy makers, hospital executives, clinicians and by patients themselves [4, 5]. Improving the ability to access, and understand, quickly visualise and compare high quality data related to patient care is known to enable improvements in health outcomes [4, 6]. However, only a minority (less than 15 %) of healthcare organisations worldwide are adept in data-driven processes to support informed decisions at point of care, as reported in a large study involving over 700 health care leaders [7]. The Learning Health System (LHS) has been proposed as a framework for incorporating best practice and taking a systems approach [3, 8–10]. The LHS involves processes that generate and apply the best available evidence and use data to support collaborative healthcare decision making with patients and clinicians. It drives the natural integration of research into high quality patient care and practice and promotes innovation, quality, safety and value in healthcare [1].

In a recent systematic review, at least 23 LHS environments were identified globally that are translating data-driven evidence into clinical practice [11]. These LHS were co-developed with many stakeholders (including multidisciplinary teams of frontline clinicians, researchers and community members), provide timely data access and analysis and require a diverse skilled workforce to make sense of the data arising from complex healthcare environments [11]. These LHS reported having direct health impact, and are therefore suggested as the necessary environments to shape the healthcare systems of the future.

In implementing complex systems level interventions, such as in a LHS, there are a myriad of frameworks and models, as well as barriers and enablers known to impact uptake in health care [1]. Key factors include leadership commitment and behaviours [12–14], technology acceptance, timing of new innovations and technology [15, 16], innovation cultures [17], cross sector integration and readiness to change [18], and occupational stress [19]. Relevant disciplines and expertise include consumer and stakeholder engagement, data technology and adoption, implementation and complexity science and health care improvement. The literature in these areas is broad, applies inconsistent terminology, encompasses overlapping theories and concepts, and often lacks focused and pragmatic insights to implementation within complex health care settings. To address the gap, in systems level approaches to the LHS, available evidence on the common elements in ‘theories/frameworks/models’ required for complex intervention implementation in health care has been summarised by Melder et al. [1]. Here the common elements required were: (1) clinician and stakeholder engagement and clinician leadership; (2) access to the best available evidence and measurement; (3) rigorous yet pragmatic processes; and (4) application of iterative models that learn from successes and failures [1].

The Australian Health Research Alliance (AHRA) is a collaboration of ten Research Translation Centres, including three Regional Centres in Australia [20] accredited by the National Health and Medical Research Council. These Centres are modelled on international Centres including Academic Health Science Centres and Applied Research Centres in the UK [21]. AHRA, across these Centres covers over 90% of government-funded health and medical researchers in Australia, and over 80% of hospitals with significant primary care engagement. AHRA has developed three priority areas for Data-Driven Healthcare Improvement activity [20]. One is to create health data hubs to stimulate partnerships across academic, clinician and other stakeholders. Monash Partners, an Australian Academic Health Science Centre in Melbourne Australia, led the development of the Learning Health System (LHS) framework through a systematic review [11] and other key literature [1], qualitative research and co-design workshop with the goal of developing a network of sustainable LHS in Australia.

Although definitions vary, including those that identify an LHS as having operational precision medicine capabilities at point of care [8], here we took a broader definition, informed by broad expert stakeholder needs across community, healthcare professionals and managers, data and information technology experts, academics and policy makers. We defined the LHS as a system in which routine data, from service delivery and patient care, can lead to iterative cycles of knowledge generation and improvement in healthcare, as a result of daily practice [22, 23]. Despite the availability of big data from healthcare, little is known about how to create an effective, sustainable LHS that stimulate partnerships across different stakeholders, to utilise data iteratively for better healthcare and health outcomes. The aim of this project was to explore core elements of a sustainable LHS to inform development of a framework for LHS in Australia.

## Methods

### Design

This was a qualitative study using semi-structured interviews. The study was methodological informed by Bhaskar’s (2008) critical realism [24]. Qualitative research is compatible with a critical realist inquiry [25] as it seeks to explain and understand complexity, and views the experiences and perspectives of people as ‘real’ [26]. The design was a purposive, qualitative study informed by an expert Academic Health Science Centre committee and a National AHRA committee, who had identified that a wealth of relevant information could be sourced from leaders who have experience of supporting or developing data innovations in healthcare nationally and internationally. The study design was also informed by the previously mentioned evidence synthesis on elements required for complex health care interventions [1] and evidence from our recent systematic literature review [11].

This study was approved by the Monash University Human Research Ethics Committee (Project ID: 19969).

### Setting

The Commonwealth of Australia has a population of 26 million and is highly urbanised with almost 90% of people residing in cities of 100,000 people or more. Australians are covered by a tax-financed universal public health insurance scheme, which provides rebates against the cost of medical fees. Private healthcare insurance is also available and the multi-payer health system is high quality but complex [27]. It is managed by different levels of Australian government (federal, state and territory, and local) and private providers to deliver (public and private) services through a range of funding and regulatory mechanisms [28]. Although complex, the Australian health system has relatively good outcomes and has been ranked as the second best health system (after the United Kingdom) by the Commonwealth Fund’s International Health Policy survey [27, 29]. However, there is room for improvement, especially as the ageing population increases, and demand on the system is predicted to exceed capacity in the coming decades [28]. Waiting times for some elective procedures in public hospitals are considered too long, with up to 15% of patients waiting over 12 months in 2015–16 [28, 30]. Inefficiency, waste and provision of low value care are recognised in the Australian health system, and are estimated to account for 30–40% of national health costs, through ineffective health interventions, administrative inefficiencies and inefficient pricing [31, 32]. Quality and safety considerations are also recognised with up to 17% of total hospital activity and expenditure related to adverse events; and one in ten patients will experience some form of harm while in the health system (such as infections, medication complications, delirium and cardiac complications) estimated to cost 9% of total hospital expenditure [32–34].

The Australian government now requires research to be embedded in health care and have charged the Academic Health Science Centres to drive this as well as establishing a National Clinical Trials Governance Framework and including research in hospital accreditation metrics [35]. AHRA Research Translation Centres as described earlier, have collectively prioritised data-driven, system-level and embedded initiatives such as the LHS, to address gaps in evidence translation, to meet government requirements and to assist health care settings to build quality research [20].

### Procedures and data collection

**Fig. 1:**
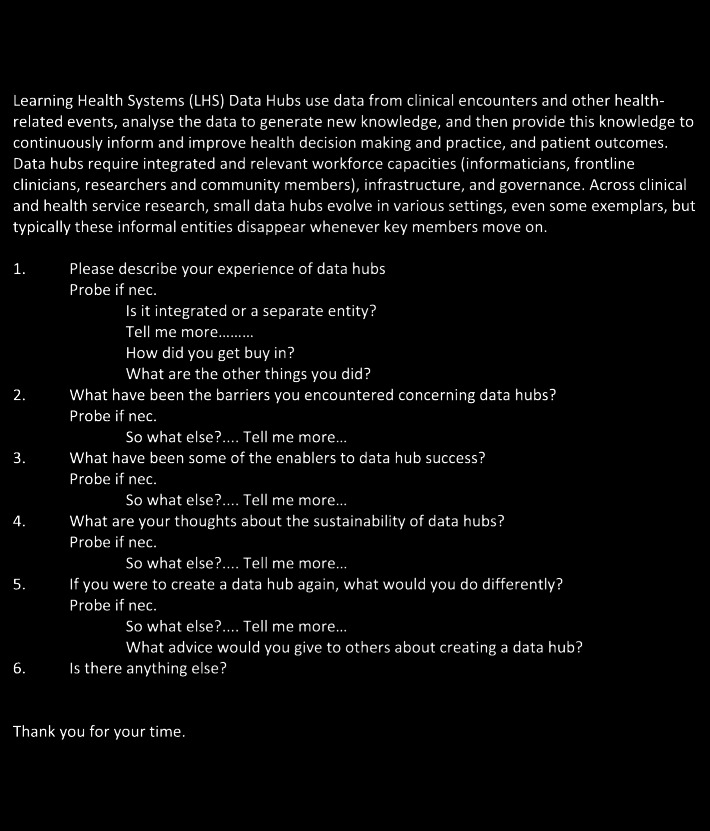
Learning Health System description and interview guide. The first paragraph containing the LHS description was read aloud to all interviewee’s at the start of each interview. This interview guide was developed for this study

A purposive sample of leaders from a range of clinical and health informatics areas, who had experience of creating or developing data hubs, were invited to take part in a semi-structured interview. The interview guide was developed for this study (Fig. 1). Leadership roles in managing data, as well as overseeing other leaders responsible for collection of clinical and health data were targeted. Purposive sampling also included those in a leadership role in healthcare delivery and/or health services research nationally or internationally. To maintain confidentiality of those interviewed, demographic and role details have been withheld.

The inclusion criteria for interviewees aligned with the above and included: 1) A nominated representative from each AHRA centre, 2) A nominated representative from each health service organisation of Monash Partners Academic Health Science Centre, and 3) international experts with published papers in LHS, or from leading organisations with known expertise in LHS or management of health related data. Nominated experts suggested by other interviewees were also considered for interview. Consumer representatives who were members of Centre data committees were also interviewed. Snowball sampling was used when a referral was suggested in a study interview. Contact details for potential participants were obtained from organisation’s websites or through referral. The only exclusion criteria was non-English speakers.

Potential participants were invited to take part in the study by an introductory email or phone call and then followed-up by one of the authors ([AJ]1) to provide study information, answer questions and organise mutually agreeable interview times. Those who agreed to participate were emailed a participant information form before the interview occurred. At the start of the interview, they were asked if they had received the explanatory statement. If they hadn’t received it, then they were then immediately emailed it directly by the interviewer. They were asked to read the explanatory statement and ask any questions. In the explanatory statement, all participants were notified that any information they provide would be confidential and any details that might identify them would not be published or made public; these points were also verbally explained by the interviewer. Participants were also advised to keep the explanatory statement. Participants were then asked if they consented to participate in the interview study and if they consented to the interview being recorded and later transcribed. Only after verbal consent was given, did the interview begin. This consent was recorded with the remainder of the interview as a record of the participant’s consent.

Twenty-eight people were invited to take part in an interview. One potential candidate declined interview and another candidate cancelled and did not reschedule. Each interview commenced with the interviewer stating the purpose of the interview and a brief description of the LHS, ascertained from a recent systematic review [11]. The interview guide covered questions about what barriers and enablers were encountered when creating or sustaining LHS environments (Fig. 1). Other questions explored what is important to ensure the sustainability of these environments, and sought insights into if they could create a LHS again, what might they do differently.

All interviews were conducted by a single interviewer who is a health services researcher trained in semi-structured interviewing techniques. Twenty-four interviews were undertaken using teleconferencing software and two face to face between 2nd July and 11th October 2019. Interviews were audio recorded and professionally transcribed. The mean interview time was 55 min. After 26 interviews, no new themes were evident and recruitment was ceased.

### Sample size

The sample size was based on the criteria outlined by Malterud (2016) [36]. This included the (a) exploratory aim of the study, (b) decision to interview targeted key informants, (c) ongoing analysis as data was collected, (d) detailed information obtained in the interviews and evidence of repetition in the dataset, and (e) employing a thematic analysis. Thematic saturation was apparent with repetition emerging during the ongoing analysis.

### Analysis

All interview transcripts were loaded into NVivo 12 (QSR International, Doncaster) for data management and coding. A thematic analysis was conducted using iterative and inductive processes following the phase of analysis outlined by Braun and Clarke [37]. An inductive approach was undertaken, as the research aim was exploratory, with the purpose of generating new knowledge.

To ensure familiarity with the dataset, transcripts were read multiple times. Coding involved generating initial codes based on content and meaning, relevant to the research question. Next, themes were searched for among the codes. Codes were then organised by separating, combining and refining to form overarching themes [37]. The entire dataset was subsequently reviewed to ensure the themes and subthemes represented a coherent pattern and to check if any additional data needed coding. Themes were then named and checked for the content captured and for the overall ‘story’ they conveyed. Data analysis was conducted by the first and third authors and discussed at regular meetings with the research team. Varying perspectives were considered and incorporated as the analysis was undertaken.

### Rigor

Records of key analytic and methodological decisions were made to ensure rigor throughout the analysis [38]. Regular consultation between the first author and third author, and meetings with the larger project team, fostered discussions about data interpretations enhancing trustworthiness. Presentations of the evolving analysis to peers and stakeholders enabled peer review to enhanced data validity [39]. A descriptor after each excerpt in the results refers to a unique participant identifier.

## Results

The 26 expert participants interviewed were nominated lead representatives from: ARHA Research Translation Centres (*n* = 10), Monash Partners Academic Health Science Centre member organisation nominees (*n* = 8), the Australian Digital Health Cooperative Research Centre (*n* = 1), State Government (*n* = 1), National Australian Digital Health Agency (*n* = 1), Public Health Research Network (*n* = 1), National Stroke Registry (*n* = 1), a consumer advocate (*n* = 1) and international experts from the UK (*n* = 1) and Canada (*n* = 1).

**Fig. 2:**
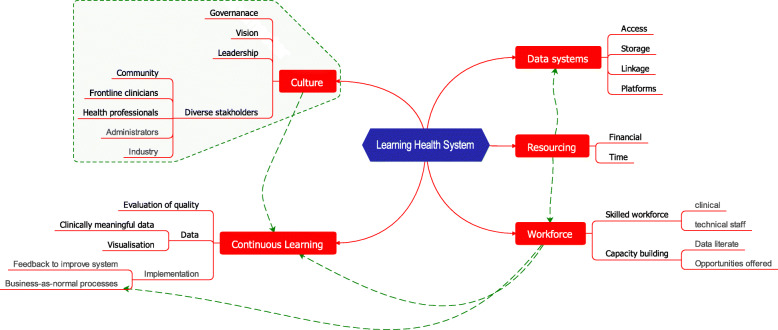
Map showing the themes and subthemes for a sustainable Learning Health System. A conceptual Map of the findings has the overarching 5 themes and subthemes underneath

Whilst clear themes were identified and little disagreement on the fundamentals emerged, there were nuances related to the role or experiences of participants. A conceptual model of the findings is shown in Fig. 2. The key themes and subthemes are summarised Table 1. Excerpts from the transcripts are provided to exemplify and illustrate the subthemes. Extra quotes from participants are found in Table 2.

**Table 1:** Themes and subthemes for a sustainable Learning Health System

Themes and subthemes:	
1	**Systematic frameworks, approaches and iterative, continuous learning with implementation of outcomes into healthcare**
	● Continuous learning
2	**Broad stakeholder, clinician and academic engagement, with collective vision and leadership**
	● Engaging diverse stakeholders from all levels and disciplines, including active health professional engagement ● Governance, structure, culture
	● Vision and leadership
3	**Skilled workforce, capability and capacity building**
	● A data literate, skilled clinical and technical workforce is vital
4	**Resources and investment over time**
5	**Data access, systems and processes**
	● Data sharing, transparent processes and consent
	● Infrastructure, data storage, timely access and streamlined data linkage and processes

**Table 2:** Table of extra quotes from participants

*ID 03: ‘You try and then you try again and then you try again and again and then you do it again and then you take a breath and then you do it again and again and again and again and again, and again and again and again and again. I’ve been doing this for 17 years now.’*	
*ID 01: ‘If you set something up and it just goes into a black hole and no one ever sees it you have problems. Not only are clinicians not interested in it, but the data you get will be rubbish. You need that loop, you need that feedback, so that the people who are entering the data can see the value of it and they start acting on it, and they start making sure the data is of a high quality when it goes in.’*	
*ID 05: ‘Strong multidisciplinary approaches are really important. And then I think obviously a really important aspect of the team is having clinicians involved.’*	
*ID 06: ‘There’s got to be initially a really clear vision about what is it you want to achieve from it, and not just in the next 2 years but the next five to 8 years.’*	
*ID 09: ‘It’s all about expert leadership.’*	
*ID 05: ‘Sometimes I will know what I want and I will be trying to say, ‘Can we do this? Can we do this this way?’*	
*ID 06: ‘And data doesn’t come for free. It’s expensive in terms of the setup of it and also the ongoing stories of that data in whatever form it is.’*	
*ID 12: ‘But right now, the situation is the funding for these things comes from multiple sources and sustainability is a major issue because people are usually unwilling to commit funding for more than a year, 2 years, 2 years and after that, they need to revisit things’*	
*ID 13: ‘It takes a brave CEO, who tends to have an appointment term of about 5 years, to invest in something that may not bear fruit for a few years.’*	
*ID 04: ‘There’s nothing more powerful than demonstrating a positive outcome.’*	
*ID 06: ‘The informatics profession is an emerging profession and is going through its own process of specialisation. Where you previously would’ve had people putting data in and then you’ve had people getting data out, there’s now a growing community. This includes data scientists, perhaps a visualisation expert, structural infrastructure expert, as well as those who design the architecture of the warehouse. There’s growing specialisation. Historically, things were pretty simple. Whereas now that’s probably about 10 different professions along that line.’*	
*ID 15: ‘Sustainability requires that this produces value which can be quantified that can then turn into profits or savings or increased values, which can go back into this spot. But it can’t be left for the people in the periphery to find that money because the people in the periphery will always be short of money and will use this money to soak up their deficits wherever they happen to have them. Has to be allocated centrally, for that purpose.’*	
*ID 02: ‘It needs to be embedded in the business that information is valuable.’*	

### Systematic frameworks, approaches and iterative, continuous learning with implementation of outcomes into healthcare

#### Continuous learning

Establishing an iterative innovation cycle early in the implementation of a LHS was identified by interviewees as important. A cycle of innovation was required to continuously drive better patient outcomes and improve services. Some participants reported that it was easy to make changes when initiating some type of quality improvement exercise, but the challenge was to make evidence based changes that were sustainable over time.

ID 01: ‘You need that loop, you need that feedback so that the people who are entering the data could see the value of it and they start acting on it and they start making sure the data is of a high quality when it goes in.’

ID 02: ‘This stuff takes time, it’s not an instantaneous improvement you’ll get, and you do need to invest in time. And it’s a bit like having to do a plan cycle; it is a continual improvement cycle. You are not going to get it right straight up.’

These key informants emphasised how a sustainable LHS requires continuous learning, needing persistence and investment of time. Further, the informants identified that a cyclic improvement process, whereby helping clinicians to see the usefulness of data outcomes for patient care, was key to ensuring high quality data was entered. As such, systems should be interconnected, iterative and cyclical.

Creating a vibrant learning culture that promoted continuous iterative learning through use of data and evidence within a LHS was noted by participants to be unsustainable without top down (e.g., CEO, leader, manager) and bottom up (e.g., clinician and consumer or patient) support. Support from all stakeholders was required to ensure continuous learning and improvements, underpinned by data and measurement. Further, a positive cultural environment was reported to foster ownership and a sense of responsibility for the quality and relevance of care and services provided.

ID 04: ‘You attract more people and more organisations and so then you create a vibrant ecosystem where this kind of activity is going on all the time’.

ID 05: ‘I think the other thing that’s key to sustainability is if it [data] is being used. So if it’s being well utilised, not just by researchers, but by the health service as well, then people will see value in it. And that will help with the ongoing funding as well.’

These key informants highlighted the necessity for organisations to adopt a ‘business as usual’ attitude to using data as part of a continuous learning cycle.

Interviewees identified that a LHS must have a framework, systems and processes to survive when inevitable changes such as staff turnover and organisational restructures occurred. It was also noted that access to routinely stored data is often tied to knowledge held by individuals, in healthcare environments, rather than at a systems level. This limits sustainability of these systems with knowledge lost with staff movement. In a LHS, underlying frameworks, systems and processes should be documented and embedded.

One informant described how a LHS could be adaptable to be robust and flexible in the face of workforce changes and to manage sustained growth into the future:

ID 06: ‘I think so far a lot of these [LHS environments] have been done on goodwill and have worked best where people who have connected well get together and do some stuff. And we’re now moving into a place, they’re getting more substantial so it needs to be in more formal structures. I think where I’ve seen previously is you need to transition from the interested parties who can work well together to then actually the formalised structure that allows it sustainability of that, regardless of those particular individuals leaving that provide the sustainability.’

### Broad stakeholder, clinician and academic engagement, with collective vision and leadership

#### Engaging diverse stakeholders from all levels and disciplines and governance

Diverse stakeholders influenced how structures were set up in the LHS. Participants recommended that governance committees should include stakeholders from clinical, community, research, human resources, finance, information technology, electronic medical record and data analytics teams. Involvement from analytics teams was considered important, as people from these teams could create meaningful knowledge from data, and use effective ways to improve data visibility. Further, participants considered the involvement of consumers as important in a LHS as they could make valued contribution to prioritising data, commenting on measures to be collected and analysed, and deciding time frames for data collection.

One informant emphasised that engaging diverse stakeholders in LHS environments was similarly important as effective governance and processes:

ID 07: ‘So you need governance, you need process, you need engagement. You need to stare down the barrel of the self-interested groups and hold them to account through consumer, health service and health professional engagement. Then, you need to convince everyone that a system like this is the only way to go.’

#### Clinician engagement and ensured inclusion

Participants stated that clinician involvement was an important resource in a sustainable LHS. However competing clinical workloads often made this challenging, as highlighted by one informant:

ID 02: ‘Our priority is patient care, and I know data does drive improvement in care and service delivery, but you are continually battling with other priorities and it’s the ability to show worth of the data.’

Clinician input into all stages of a LHS from engaging in the vision, to setting clinical priorities, to bringing in expertise and evidence, to analysing data and implementing this back into practice improvement, was seen as important. A LHS was necessary to evaluate and optimise the quality and accuracy of the data, use of data and to implement and drive improvements. Interviewees expressed that clinician buy-in would not be possible without protected time to contribute to the LHS as most clinicians were already operating at capacity. For example, without additional resources and data entry being an integrated and useful component of workflow, the quality of data entered was likely to be poor.

ID 05: ‘Strong multidisciplinary approaches are really important. And then I think obviously a really important aspect of the team is having clinicians involved.’

ID 08: ‘We showed you could get the clinicians in the hospital together, they look at the gaps in care, ‘Ok, we missed out on people getting thrombolysis’ or ‘We’re not meeting the indicator for patients with a care plan. What can we do about it?’ And they get together and they look at their gaps in their quality of care. And then an external facilitator comes in and they do a workshop and they develop an action plan of how they can improve those processes. And by getting them all in together and getting buy-in that does actually improve care. And so we’ve found with the [data-driven project], it improves care by 14% over the time period.’

These key informants highlighted the requirement for clinician involvement in all stages of a LHS to improve health care.

#### Vision and leadership

Participants stated that stakeholder engagement, collective vision and leadership were all important factors in a sustainable LHS. Developing a common vision informed by all stakeholders was essential for the coordination of multiple resources and for targeted approaches to achieve that vision. Some participants recommended that agreed priorities should be set and worked towards.

ID 07: ‘You need a vision that everybody has. That needs to be co-designed, which means we need all the key stakeholders in the room.’

ID 05: ‘If you can start with projects that are important to the health service and showcase what you can do with their data, that perhaps they’re not currently able to do, that could be a good example to use to support the LHS.’

These above informants described that to showcase the LHS approach, it is recommended to have a clear and shared vision, and start with agreed vision statements is likely to optimise prompt and positive change.

While consensus on a collective vision was crucial, leadership actively committed to the vision was also identified as important. Leadership and strategic planning to embed appropriate processes and systems, was also reported as essential for creating a sustainable LHS.

ID 08: ‘Leadership is really important. If you’ve got really good leaders and they can get really good buy in …*. ’.*

ID 10: ‘The key enabler is leadership in my view – leadership that sets the direction, frames what you are trying to do and then brings people along. You will need some smart people, you will need some money to cover salaries and you will need some good technology.’

The above informants describe leadership as necessary to ensure resources were available and directed towards supporting the vision, to foster the buy-in from multiple stakeholders.

Leadership to cultivate trusting relationships between clinicians, other healthcare staff and managers was also reported as important aspect of a LHS. A sustainable LHS requires leaders to facilitate the agreement of measured outcomes, and to foster trust that these measures will be used for the agreed purposes and not for penalising individuals or the withdrawal of funding. This informant highlighted trust as important:

ID 03: ‘Trust is the barrier, if you don’t trust what data is being used appropriately. People get pretty tired, particularly in hospital settings, of data being used as a club, ‘You’re not hitting your four hour targets, bastards’

### Skilled workforce, capability and capacity building in a sustainable LHS

#### A data literate, skilled clinical and technical workforce is vital

An important resource in a sustainable LHS identified by participants was data literate healthcare and technical staff. Data skills and knowledge identified as important for healthcare staff included familiarity with data collected, and understanding and interpreting data.

ID 02: ‘I think we have a real skill shortage. How we address that, I am not sure. I think that the skill shortage is in the technical fields, and also in the clinicians that work on the ground. If we are moving towards more data-driven improvements and using data to help with workflows or patient flows, clinicians need to understand how to use the data. How do you drive improvements through the use of data?’.

This informant highlighted the necessity for data-literate staff and also the need to upskill many current healthcare staff with data acquisition, analysis, knowledge generation using data and how it can drive better outcomes.

Further, participants suggested that a sustainable LHS has people with highly developed data management and analysis skills embedded at various levels in a LHS.

ID 11: ‘There needs to be a core group of people who are well versed in several disciplines and that might mean handling of large data, informatics, computing, information technology people, machine learning and other similar related initiatives, data visualisation, the whole lot of infrastructure that comes with it; people infrastructure. And the trick will be to develop at least a core group who are constant and that’s where the sustainability financial need will be critical.’

This informant identified that people with skills related to data management, linkage, analysis and the different ‘languages’ from related disciplines must be available in a LHS for growth and sustainability.

Collaboration between data specialists, clinical staff and academics was seen as beneficial in a LHS. University academics specialising in IT and statistical analyses were important resources that could provide external expertise when required.

ID 02: ‘There needs to be really good relationships and partnership between the clinical and the data people. Because I think with that a symbiotic relationship, they’re both dependent on each other. The clinical people are dependent on the data people to get it out and make sure it’s the right thing and the data people were reliant on the business to actually run with it, know their own business, and understand how it can drive improvement.’

ID 11: ‘Not one hub can do everything by itself; the one hub will have buy-in from related organisations industry, university organisations.’

These key informants underscored the importance of developing connections and relationships to facilitate a sustainable LHS.

### Resources and sustainable funding

A key resource for creating a sustainable LHS was guaranteed funding and long-term investment. Costs were incurred as implementing a new LHS into a health system already running at capacity requires a coordinated and resourced approach. Additionally, costs were associated with obtaining, storing, analysing and using data.

ID 02: ‘You need funding. It needs to be funded. And not a one-off funding but recognition that data changes and these types of hubs need some degree of maintenance as well, so there’s the technical and obviously the workforce element to be able to pull the data out, and protect it.’

ID 14: ‘I want to create a structure that allows us to be a unit that has longevity and sustainability. So I’d really like to move towards three to five year plans where we can put data scientists in that aren’t pre-committed to part of individual projects but can help build a resource to allow you to have a genuine hub.’

These key informants identified protected longer-term funding, was key to the viability of a LHS, as the resources/investment to establish a LHS were required over a number of years before benefits were seen, involving significant cost.

### Data systems, processes, access and use

#### Data sharing, transparent processes and consent

Transparent governance, systems, structures, agreements and clarity of roles, responsibilities and activity within the LHS were all seen as vital for its continued success. This informant described the importance for clear data access and consent processes.

ID 05: ‘We need to be more and more transparent around how to use people’s personal data and having community education and transparency. That is, I think, very important.’

Participants perceived a key part of sustainable a LHS was transparent legislation that informed how patient data would be used within the system to create better healthcare. Timely access to data, information and reports was essential to a LHS. If layers of patient consent, agreements, permissions and sign offs resulted in major delays, then the LHS would not be sustainable. This informant called attention to system efficiency as a key LHS component.

ID 12: ‘Make a learning health system function efficiently, within the workflow of an organisation, so all of the necessary approvals are done with the least amount of pain.’

#### Infrastructure, data storage, timely access and streamlined data linkage and processes

Interviewees identified that there are available affordable, secure, accessible and integrated systems for data storage. It was stated that there are many options available in the current market and that emerging LHS environments would use existing infrastructure and technologies. Timely access to relevant data was reported as fundamental to sustainable LHSs. One informant described access to data for informed decision making in real-time was pivotal in a LHS:

ID 04: ‘We want to move more towards identified, real time and live data that can be used to help patients, clinicians and researchers.’

Hospitals already generate mandated reports for the Government such as length of stay, waiting time in ED, and number of re-admissions. Participants identified that data linkage was important for producing information about quality, models of care, and health outcomes. Several participants mentioned a desire to link health data to other relevant data. Health and healthcare does not exist in an isolated bubble. The social determinants of health are particularly relevant when evaluating value based care or health outcomes when taking into consideration a wider social picture rather than a siloed health perspective. It was expressed that there is much that could be learnt through the linking of health data with that for housing, pensions, education, and crime and corrections data. However, current data linkage processes are often drawn out and convoluted and therefore unfavourable for current LHS environments in Australia. One participant suggested that streamlining federal and state based legislation would facilitate data linkage and more timely access to various data sources:

ID 01: ‘So many things have been duplicated across Australia, every state is doing its own thing; it’s so inefficient.’

A sustainable LHS efficiently produces practical, informative and up to date data. This participant described how data from a LHS could be strategically used and the subsequent benefits:

ID 01: ‘Now the beauty of it was that suddenly not only could you follow what was happening in real time in your department, but you could also interrogate and get all sorts of stats and there are all sorts of standard tables that they had, there were reports that would spit out. Using this reporting system, we could also refine the reports even more. You could also set up triggers, so - whether it was at triage or with the nurse inside with the doctor - when they put in certain words you could set it up so up come half a dozen questions as a result. So suddenly it just transformed the way that we were doing business.’

The importance of a culture and processes that make the integration of data, information and research, routine in healthcare delivery was important in a LHS. For this approach to be sustainable in a LHS, organisational level support was seen as essential.

ID 02: ‘this is about improving clinical care by getting organisations to understand the use of their data and the benefits of using their data, in many ways much more than it is about the academic use of the data.’

ID 08: ‘We want to be more internally-driven so that internal facilitation, the quality managers in the hospital look at these live reports and say, ‘How can we improve that in our hospital rather than externally driven?’

These informants conveyed the importance of embedding systems in the culture of an organisation to automate data retrieval and reports which included benchmarking.

## Discussion

There is a requirement for transformation in healthcare to deliver better health outcomes. Digital health data has the potential to underpin this transformation towards more efficient and effective services. This project leveraged multidisciplinary leaders nationally and internationally to explore core elements of a sustainable LHS and inform development of a LHS framework for the Academic Health Science Centre context in Australia. Key themes were identified including the need for systematic approaches and iterative, continuous learning with implementation of outcomes into healthcare; broad stakeholder, clinician, academic and community engagement with collective vision, leadership, governance and trust; skilled workforce, capability and capacity building; resources in human capital and investment, and; data access, systems, standards and processes.

A major theme identified was that a sustainable LHS requires systematic frameworks, approaches and iterative, continuous learning with implementation of outcomes into healthcare. Planning to embed processes into usual practice to capture and use data and learnings was key to success, consistent with literature on registry based LHS [4, 6]. Ad hoc LHS-like environments were acknowledged as common in healthcare, but participants agreed that these were unsustainable and waned, when key personnel moved on. These offered little for those at the frontline in terms of tangible healthcare improvement, yet frontline engagement was vital for success and sustainability. Transparent and iterative, cyclical improvement processes in the LHS to enable timely access, sharing and streamlined linkage of data, with mechanisms to feedback into frontline care, were recognised as important in ensuring the LHS approach was impactful and sustainable. These findings are commensurate with the emerging literature that describes systematic approaches and processes that are common in all LHS environments; see further below for more discussion about this [1, 2, 9, 11, 40–42].

Broad stakeholders including health professionals, managers, academics and community engagement was deemed important. Indeed partnering with all people who contribute to a healthy LHS including patients and the wider community, alongside all staff working in healthcare, was prioritised. The continuous learning within a LHS was dependent on diverse groups engaging and working together to innovate and solve complex healthcare problems around a shared vision. In this context, strong credible leadership, identification and commitment to a collective LHS vision was important. Transparent governance and clear structure was identified as a sub-theme. Collecting and storing data, without clear purpose or vision, was felt to contribute to lack of sustainability, because the worth of the data and interactive cyclical improvement benefits were not seen. This is worthy of consideration when a LHS is focused on data use only for research or health service management, rather than extension into improved clinical care. These views are in line with the vast literature on health care improvement and implementation science research [1, 43, 44], and also highlight the importance for improvement in complex health systems to assist clinicians and others to engage and lead change [1].

Skilled workforce, capability and capacity building in a sustainable LHS was another major theme. A data literate, skilled clinical and technical workforce, with access to education and training was reported as key to a LHS. Highly developed data management and analysis skills in staff embedded at various levels was also recognised. Collaboration between data specialists, clinical staff and academics was seen as beneficial and a strength of an LHS. University academics specialising in IT and statistical analyses were seen as important resources and can provide external expertise when required. These skills and resources in human capital underpin a sustainable LHS and also should be iteratively enhanced and expanded over time. This is supported by a recent systematic review that identified health improvements arising within 23 LHS environments, and all had combined people with relevant workforce capacities and people with data analytic capabilities to make sense of the complex data arising from complex improvement cycles to address areas of unmet need, public interest and priorities [11].

Other resources and investment were also noted as essential in building a sustainable LHS. In addition to investing in workforce, there are costs associated with obtaining, storing, analysing and using data. It was reported that it takes significant resources and protected funding, sustained for a number of years to establish a LHS and often this investment is required before benefits emerge. An essential component of a LHS is a collaborative platform that provides connectivity across silos, organizations, and professions. In the literature there is evidence that automated reports using the data from the entire LHS has led to the efficient identification of patients for standardised care, specialised care, follow-up or clinical trials [45–47]. Collection of information directly from patients before the clinical encounter has also been reported as beneficial due to time efficiencies [48], as well as creating PROMs (patient reported outcome measures) that are saved within the electronic medical record and can enable longitudinal tracking of individual patient outcomes and are used in aggregated research [4, 5, 48]. There are also examples of existing LHSs that invested infrastructure to enable patients to self-track their condition [4, 5], for example the electronic patient-reported outcome measurement system to identify distress and despair in cancer patients, which then lead to more referrals to psychological care [5].

Data access, systems and processes to producing useable, useful and quality data was identified as a theme and is generally reported in literature on the impact of LHS models [3, 8–11]. We found that appropriate infrastructure and transparent processes are required to operationalise data access, linkage, analysis and applications. Participants clearly expressed that from the patient perspective, there is a responsibility to ensure their data can be compiled, accessed and analysed to benefit their own health and that of the broader community. This requires a culture of trust, transparency, partnership and co-design. For example, a Swedish system enables a patient to record symptoms, health status, and quality of life directly into their electronic medical record. Patients access their record at a clinic or at home. The LHS combines their data with other data (clinical examinations and laboratory results) in graphical displays of health status over time. The patient and clinician can view this together, or separately, and this helps patient and clinician partnerships to optimize health. Data is also exported into the national registry, enabling research and ongoing learnings, contributing to improving population health. Evaluations have found that patients greatly value this system [4, 6].

### Connection to extant literature

These findings are commensurate with the broad literature that describes systematic approaches and processes required for system-level complex interventions in health care, with our research suggesting this is also required for a functioning LHS [1, 2, 9, 11, 40–42]. As recently summarised by Melder et al. [1], such complex interventions require rigorous yet pragmatic processes and iterative applications that learns from success and failures and drives health care improvements [1]. This continuous learning culture was identified as our first key theme. Skilled workforce, capability and capacity building was identified as another of our key themes and these are essential to power the rigorous (yet pragmatic) processes to drive the LHS continuous learning culture.

Clinician and stakeholder engagement and clinician leadership is another recognised element in the literature on complex interventions in health care [1]. This aligns with our second theme: broad stakeholder, clinician and academic engagement, with collective vision and leadership. Trust was identified as a sub-theme, which resonates with trust appearing in a number of leadership models [13, 14] and trust in technology has been found to be influential in organisation wide adoption studies [49].

Access to the best available evidence and measurement is also purported to be required in the literature about complex interventions in health care [1]. This was captured in our key themes of ‘Data access, systems and processes’ and ‘Resources and investment over time’.

### Limitations

The semi-structured interviews were informed by a national steering committee in data-driven healthcare improvement and international systematic literature review. The nature of the questions may have influenced the breadth or responses provided. We aimed to inform development of LHS in the Australian context and hence interviews from Australian experts were preferenced and results may not be generalisable. Overall, diverse stakeholders were interviewed and thematic saturation was reached, however additional stakeholders and stronger consumer and international participation may have influenced the outcomes.

## Conclusion

The themes identified in this qualitative study in data-driven healthcare improvement, provide insights into the core elements in a sustainable LHS. Fundamentally, an LHS requires continuous learning, generating evidence for implementation back into frontline care to improve outcomes. Structure, governance, trust, culture, vision and leadership were all seen as important, along with a skilled workforce and sustained investment. Processes and systems to optimise access to quality data were also seen as vital in an effective, sustainable LHS. These findings will inform a co-designed framework for implementation within the Australian Academic Health Science Centre context. Application of these findings, offers the potential to accelerate the use of health data to iteratively produce new knowledge to in-turn improve healthcare delivery and health outcomes.

## Data Availability

The datasets used and/or analysed during the current study are available from the corresponding author on reasonable request.

## Abbreviations

LHS: Learning Health System
AHRA: Australian Health Research Alliance
AHRTC: Advanced Health and Research Translation Centres
CIRH: Centre for Innovation in Regional Health
